# A frictional soliton controls the resistance law of shear-thickening suspensions in pipes

**DOI:** 10.1073/pnas.2321581121

**Published:** 2024-04-16

**Authors:** Alexis Bougouin, Bloen Metzger, Yoël Forterre, Pascal Boustingorry, Henri Lhuissier

**Affiliations:** ^a^Aix Marseille Univ, CNRS, Institut Universitaire des Systèmes Thermiques et Industriels, Marseille 13453, France; ^b^Chryso France, Sermaises 45300, France

**Keywords:** pipe flow, shear-thickening suspension, jamming, soliton, friction

## Abstract

Pipe flows are commonly found in nature and industry as an effective mean of transporting fluids. They are primarily characterized by their resistance law, which relates the mean flow rate to the driving pressure gradient. Since Poiseuille and Hagen, various flow regimes and fluid rheologies have been investigated, but the behavior of shear-thickening suspensions, which jam above a critical shear stress, remains poorly understood despite important applications (e.g., concrete or food processing). In this study, we build on recent advances in the physics of shear-thickening suspensions to address their flow through pipes and establish their resistance law. We find that for discontinuously shear-thickening suspensions (large particule volume fractions), the flow rate saturates at high driving stress. Local pressure and velocity measurements reveal that this saturation stems from the emergence of a frictional soliton: a unique, localized, superdissipative, and backpropagating flow structure coexisting with the laminar frictionless flow phase observed at low driving stress. We characterize the remarkably steep effective rheology of the frictional soliton and show that it sets the resistance law at the whole pipe scale. These findings offer an unusual perspective on low-Reynolds suspension flows through pipes, intriguingly reminiscent of the transition to turbulence for simple fluids. They also provide a predictive law for the transport of such suspensions in pipe systems, with implications for a wide range of applications.

## Significance

Pipe flow is an emblematic configuration in fluid mechanics, the basis for describing the laminar/turbulent transition, and a widespread fluid transport mode in nature and industry. Yet, it is still not known how a suspension of small particles, which shear-thickens and jams, can flow in such a confined space. We show here that it does so by nucleating a superdissipative local flow structure: a frictional soliton, which acts as a sharp flow-limiter, governing the global resistance law of the pipe. These results uncover a highly unconventional picture of viscous (low Reynolds number) pipe flows—a foundational step in improving many industrial processes.

In 1840, Poiseuille and Hagen (1–3) established experimentally the laminar resistance law for a Newtonian liquid, $Q\propto -R^{4}\nabla P/\eta_{l}$, by which the volume flow rate Q increases with pipe radius R and pressure gradient ∇P, and decreases with liquid viscosity $\eta_{l}$. Since then, a vast literature initiated by Reynolds (4) has documented the limit of this laminar regime, beyond which, inertial eddying flow structures (turbulent puffs and slugs) form and the hydraulic resistance starts deviating from Poiseuille law (5–10). Investigations have also concerned more complex materials (elasto-visco-plastic fluids or particulate suspensions), for which the effective rheology or interphase flow may modulate Poiseuille law (11–14) or alter the transition to turbulence (15–19). By contrast, the case of shear-thickening suspensions has received much less attention (20), and it is still unclear how these suspensions flow through pipes, even though they are frequently encountered in industry, from high-performance concretes (21) to food (22), and in nature, from diseased blood (23) to crystal-rich lava (24, 25).

The problem is rather general since most suspensions of small (∼5 to 50μm) nonaggregating particles actually show a steep shear-thickening behavior controlled by the shear stress level. At sufficiently large particle volume fractions, these suspensions flow under a mild stress, but discontinuously shear-thicken or jam, above a critical stress. This peculiar rheology has been shown to stem from a change in the effective friction coefficient between particles, as their mutual repulsive force is overcome (26–28). On a macroscopic scale, it has also been shown to promote new flow instabilities (29–34), complex transient flow structures (35–39), and nontrivial drag laws (40, 41), in a few common flows (in Couette cells, down inclines, or past cylinders). These jamming structures raise questions about how shear-thickening suspensions can actually flow in highly confined wall-bounded configurations. In particular, it is currently not known how they flow through a pipe, where bulk incompressibility imposes uniformity of the flow rate along the pipe.

The present work tackles this question on experimental grounds. We use a gravitational pipe drainage protocol offering a precise control on the key parameter of the flow: the mean shear stress at the wall. Near-wall visualizations combined with local and global hydraulic resistance measurements reveal the fascinating way by which the flow proceeds: At high stress, the flow nucleates a solitary backpropagating flow phase, with a plug-like velocity profile. We characterize the flow bifurcation, identify the frictional and superdissipative nature of the soliton, and show how it sets the resistance law of the suspension along the whole pipe. Last, we address the universality of the phenomenology for discontinuously shear-thickening suspensions and discuss the mechanism behind the superdissipation.

## Results

### Experimental Set-Up.

**Fig. 1.**
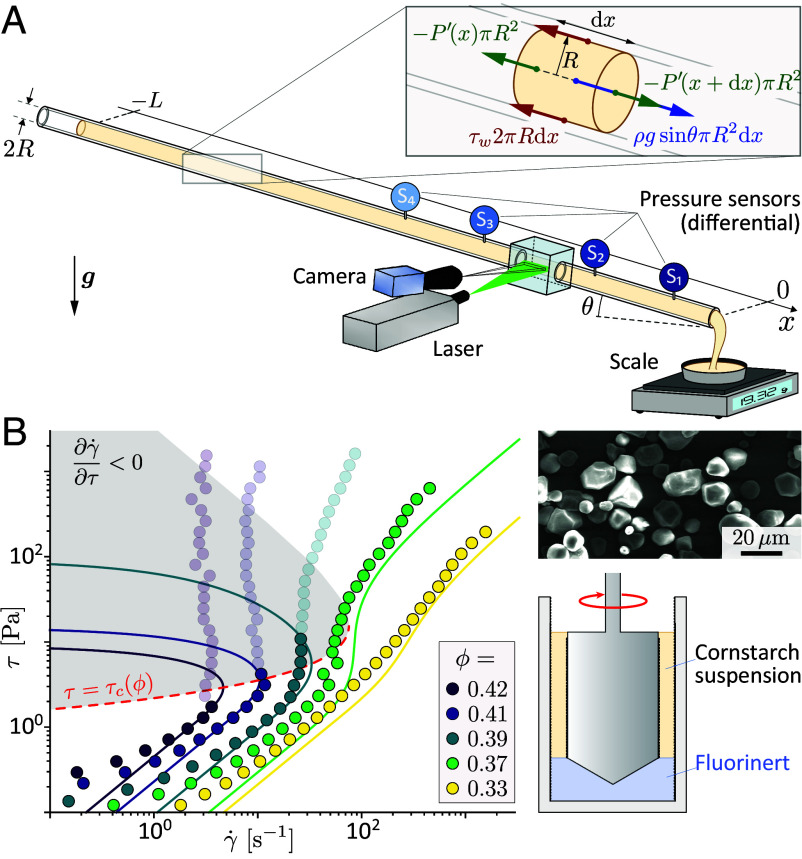
(*A*) Sketch of the gravity-driven setup allowing to control the mean wall shear stress $\left\langle \tau_{w}\right\rangle$ through pipe inclination, measure global flow rate and local bulk pressure, and visualize near-wall flow. (*Inset*) Force balance on a slice of suspension. (*B*) Suspension rheology ( 70): shear stress versus shear rate for aqueous suspensions of cornstarch grains (optical microscope image) at various solid volume fractions ϕ obtained with a cylindrical Couette cell (sketch). Solid lines: fit to Wyart–Cates rheological model $\tau =\eta_{S}{\left(\phi_{0}-\left(\phi_{0}-\phi_{1}\right)e^{-\tau^{*}/\tau }-\phi \right)}^{-2}\dot{\gamma }$ setting the viscosity prefactor $\eta_{S}=0.28\;$ mPa s, the frictionless and frictional jamming volume fractions $\phi_{0}=0.445$ and $\phi_{1}=0.385$, respectively, and the short-range repulsive stress scale $\tau^{*}=8\;$ Pa (semitransparent symbols are not used to fit). Red dashed line: critical stress $\tau_{c}\left(\phi \right)$ at which Wyart–Cates curves become negatively sloped ($\partial \dot{\gamma }/\partial \tau <0$, gray region).

To study the intrinsic pipe flow, without pumping fluctuations or entrance effects, we use the configuration sketched in Fig. 1*A*: the gravitational drainage of a long inclined pipe, initially filled with a shear-thickening suspension (see *M&M* for details on setup, materials or protocol). This configuration has two crucial advantages. i) It avoids convergent/divergent flow sections (20), which could localize stresses and trigger jamming (see experiments and discussion on convergent inlet in *SI Appendix*, SI.2). ii) Gravity ensures a steady and controllable average forcing of the flow. Indeed, since inertial effects are small (Reynolds number ≲10 with slowly varying flow), the force balance over the flow length L implies that the mean shear stress at the wall $\left\langle \tau_{w}\right\rangle \equiv \frac{1}{L}\int_{0}^{L}\tau_{w}dx=\rho gR\sin\theta /2$ is constant for the whole drainage and is simply set by the suspension density ρ, gravity g, the inner pipe radius R and the pipe inclination θ (Fig. 1*A*, *Inset*).

Most experiments are performed with an aqueous suspension of cornstarch grains (Fig. 1*B* image). This widely documented system is known for its marked discontinuous shear-thickening rheology, which we characterize at different solid volume fractions ϕ with a Couette cell apparatus (other particles are used to verify that results are generic of shear-thickening suspensions, see below and *M&M*). Rheograms are fitted with Wyart–Cates model (27) (solid lines in Fig. 1*B*) to obtain the low-stress (frictionless) viscosity $\eta_{0}\left(\phi \right)=\eta_{S}{\left(\phi_{0}-\phi \right)}^{-2}$ and the critical shear stress $\tau_{c}{\left(\phi \right)\equiv \tau |}_{\partial \dot{\gamma }/\partial \tau =0}$ (red dashed line), above which suspensions with a sufficiently high particle volume fraction ($\phi >\phi_{\;\text{DST}}\approx 0.37$) show a discontinuous shear-thickening (see caption of Fig. 1 for the definition of $\eta_{S}$ and $\phi_{0}$).

### Saturation of the Flow Rate.

**Fig. 2.**
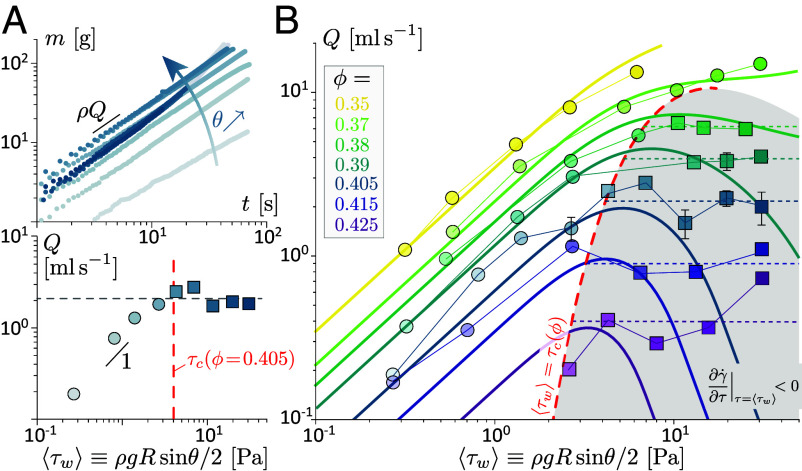
Global resistance law ( 70). (*A*) *Top*: drained mass of suspension vs time for fixed volume fraction ($\phi =0.405>\phi_{\;\text{DST}}$) and increasing pipe inclination. *Bottom*: corresponding flow rate Q vs mean wall stress. (*B*) Q for various volume fractions. Red dashed line: flow rate saturation onset criterion $\left\langle \tau_{w}\right\rangle =\tau_{c}\left(\phi \right)$, for the Wyart–Cates rheological laws fitted in Fig. 1*B*. Dashed lines: experimental average highlighting flow rate saturation in the gray region (where $\partial \dot{\gamma }{/\partial \tau |}_{\tau =\langle \tau_{w}\rangle }\;<0$, for a laminar flow). Solid lines: flow rate expected for a steady laminar flow (see also *SI Appendix*, SI.3). Symbol shape indicates experimental observation of the laminar phase only (○) or laminar phase + frictional soliton (□). R=5.15mm in (*A*) and (*B*).

We investigate, first, the global resistance law of the pipe, i.e., the evolution of the flow rate with the forcing, starting with a discontinuously shear-thickening suspension ($\phi =0.405>\phi_{\;\text{DST}}$). The forcing $\left\langle \tau_{w}\right\rangle$ is varied through the pipe inclination and flow rate is obtained by weighing the drained suspension at the pipe outlet (Fig. 1*A*). As shown in Fig. 2*A*, the drained mass m increases linearly with time over the whole range of inclination $0.5^{{}^{\circ}}<\theta <90^{{}^{\circ}}$ (i.e., 0.27 Pa $\leq \langle \tau_{w}\rangle \leq$ 31 Pa), which indicates the flow rate $Q\equiv \dot{m}/\rho$ does not vary during drainage. However, Q depends on inclination, with two strikingly different trends. At low forcing ($\langle \tau_{w}\rangle ≲$ 4 Pa), flow rate increases quasi-linearly with $\left\langle \tau_{w}\right\rangle$, as for the laminar flow of a Newtonian liquid. By contrast, for higher forcings ($\langle \tau_{w}\rangle ≳$ 4 Pa), the flow rate is found to saturate: the same value (Q≈1.8ml/s) is obtained over a more than 10-fold increase in the forcing. Notably, the mean wall stress at the onset of saturation closely matches the discontinuous shear-thickening onset stress $\tau_{c}\left(\phi =0.405\right)\approx 4.0$, as obtained from the rheological characterization (Fig. 1*B*).

To confirm and generalize these two global responses of the flow, we extend experiments to a wide range of particle volume fraction (0.35≤ϕ≤0.425) and recover that flow rate saturates at high forcings ($\left\langle \tau_{w}\right\rangle >\tau_{c}\left(\phi \right)$), provided volume fraction is sufficiently high ($\phi >\phi_{\;\text{DST}}$). The region of flow rate saturation (highlighted with horizontal lines) coincides with the region where $\partial \dot{\gamma }{/\partial \tau |}_{\tau =\langle \tau_{w}\rangle }<0$ (highlighted in gray). This confirms that flow rate saturation is triggered by the discontinuous shear-thickening of the suspension at the pipe wall, where the shear stress is maximal for a laminar flow.

This indicates that available S-shape rheological models, which capture the dependence of $\tau_{c}$ with ϕ, are sufficient to capture the onset of flow rate saturation. However, such models do not explain, alone, why and how the flow saturates. Indeed, the base-state flow rate predicted from these rheological laws for a steady laminar flow does not plateau but, actually, decreases strongly at high forcings (see solid lines in Fig. 2*B*, and *SI Appendix*, SI.3, for the derivation). Therefore, additional information on the structure of the flow is needed to elucidate the mechanism behind flow rate saturation.

### Evidence of a Localized Flow Structure: The Frictional Soliton.

**Fig. 3.**
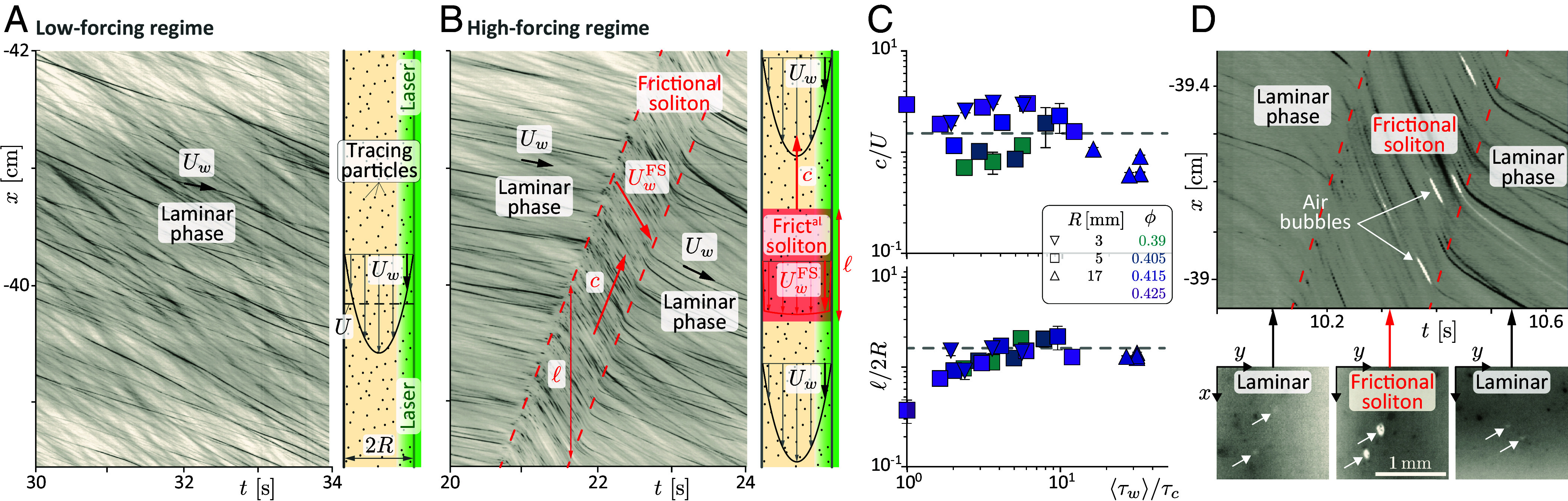
Identification of the frictional soliton. (*A* and *B*) Spatiotemporal images of the near-wall flow along a pipe generatrix, at ≈40 cm upstream the pipe outlet ($\phi =0.405>\phi_{\;\text{DST}}$, R=5.15 mm, see *SI Appendix*, Movies in SI.1). The (x,t) trajectory of the tracing particles (black) indicates the near-wall velocity $U_{w}$ [different velocities at a given (x,t) reflect different distances of the tracing particles to the wall]. (*A*) At low forcing ($\theta =4.9^{{}^{\circ}}$, $\left\langle \tau_{w}\right\rangle =2.6\;\;\text{Pa}<\tau_{c}$), a single laminar phase of steady, uniform and low near-wall velocity is observed ($U_{w}\approx 0.10U$). (*B*) At large forcing ($\theta =22.0^{{}^{\circ}}$, $\left\langle \tau_{w}\right\rangle =11.6\;\;\;\text{Pa}>\tau_{c}$), a localized frictional-soliton phase of high wall-velocity ($U_{w}\approx 0.6U$) separates two laminar phases and propagates against the flow. Sketches: Inferred velocity profiles in the laminar and frictional-soliton phases. (*C*) Scaled velocity and width of the soliton vs scaled mean wall stress ( 70). The dashed lines are c/U=1.5 and ℓ/2R=1.5. (*D*) *Top*: Spatiotemporal image of the near-wall flow highlighting the liquid pressure drop in the frictional soliton through the expansion of small probe air bubbles (white) transported by the suspension (ϕ=0.39, see *SI Appendix*, Movie in SI.1). *Bottom*: Snapshot images showing the same two bubbles upstream (*Left*), inside (*Middle*), and downstream (*Right*) the soliton.

To obtain such information we use a transparent pipe, seed the suspension with fluorescent tracers, shine a laser sheet through the wall and optically monitor the flow over a ∼8R-long pipe section located ≈40 cm upstream the pipe outlet (see Fig. 1*A*, *SI Appendix*, Movies in SI.1 and more details on optical measurements in *M&M*). Given the suspension opacity, light only penetrates a thin layer of suspension (with an estimated thickness ∼100 μm, see *SI Appendix*, SI.4), which gives access to the near-wall velocity of the suspension (see sketches in Fig. 3 *A* and *B*).

As shown in Fig. 3, the spatiotemporal evolution of the near-wall flow differs qualitatively between the low and high-forcing regimes. For low forcings ($\left\langle \tau_{w}\right\rangle <\tau_{c}\left(\phi \right)$, Fig. 3*A*, *SI Appendix*, SI.1Movie S1), the near-wall flow is uniform and steady, which is consistent with a Poiseuille-like laminar flow in the pipe, as expected from the quasi-Newtonian behavior of the suspension at low stresses. The mean near-wall velocity $U_{w}\approx 0.10U$ (averaged over the observation depth, see *SI Appendix*, SI.4), is approximatively one decade smaller than the mean-flow velocity $U=Q/\pi R^{2}$. By contrast, at high forcings ($\left\langle \tau_{w}\right\rangle >\tau_{c}\left(\phi \right)$, Fig. 3*B*, *SI Appendix*, SI.1 and Movie S2) the near-wall flow reveals an intriguing flow structure. While the near-wall flow (within the camera field of view) is initially steady and uniform, as in the low forcing regime, a short, nonsteady, nonuniform region is observed, which propagates upstream with a constant velocity c and a preserved length ℓ, before the flow returns to its previous laminar (frictionless) state. This reveals that flow rate saturation is associated with the inception of a new flow phase: a localized, purely propagative structure, which we call frictional soliton.

Near-wall velocimetry also provides information on the cross-sectional velocity profile within the different flow phases. Upstream and downstream from the soliton, the near-wall velocity ($U_{w}\approx 0.06U$ and 0.13U, respectively) remains one order of magnitude below the mean velocity U, which suggests the flow profile is Poiseuille-like, as in the low-forcing regime. However, in the frictional soliton, the near-wall velocity $U_{\;\text{w}}^{\;\text{FS}}\approx 0.6U$ has typically the same magnitude as U, which suggests a velocity profile closer to a plug flow, as schematized in Fig. 3*B* (additional measurements of the velocity variations across the near-wall observation depth also suggests a finite slip velocity at the wall, see details in *SI Appendix*, SI.4).

To document the soliton characteristics and their dependence to the suspension and flow conditions, we perform systematic near-wall observations, for varied flow forcings, particle volume fractions, and pipe radii. Measurements (Fig. 3*C*) reveal that the length ℓ=(1.5±0.5)2R of the soliton is essentially set by the local length scale of the pipe, namely its diameter 2R with, however, a mild trend toward shorter solitons close to the onset forcing ($\left\langle \tau_{w}\right\rangle ≲3\tau_{c}$), where solitons are sometimes found to be evanescent. Moreover, the (upstream) propagation velocity of the soliton, c=(1.5±1)U, is found to be of the order of the mean flow velocity, regardless of applied stress or particle volume fraction. This suggests a different mechanism from those reported for shear-jamming fronts (35, 42–44), whose propagation velocity is typically one order of magnitude larger than the flow velocity and strongly depends on volume fraction.

A crucial piece of information about the frictional soliton is also provided by observing microscopic air bubbles, which are seldomly and fortuitously trapped in the suspension. Fig. 3*D* shows the behavior of such bubbles as the frictional soliton passes. Upstream of the soliton, bubbles (tiny white spots) have a stable size of approximately 20 μm. Within the soliton, however, they grow notably, increasing their diameter by a factor 2 to 4, before collapsing back to their original size once the soliton has passed. This transient expansion of the bubbles reveals a pressure drop in the liquid of the suspension, inside the soliton. This pore pressure drop actually reflects a symmetric increase in the particulate pressure, i.e., the pressure supported by contacts between grains and between grains and the pipe wall (45–49). The magnitude of the bubbles expansion (1 to 2 decades in volume) suggests a pore pressure drop by typically one atmosphere or more (see details in *SI Appendix*, SI.5), i.e., a massive increase in the granular pressure at the wall, which controls the shear stress of the suspension. This observation suggests that the frictional soliton is the locus of a strong resistance to the flow, which calls for a more direct estimation of the stress distribution along the pipe.

### The Frictional Soliton Is a Superdissipative Structure.

**Fig. 4.**
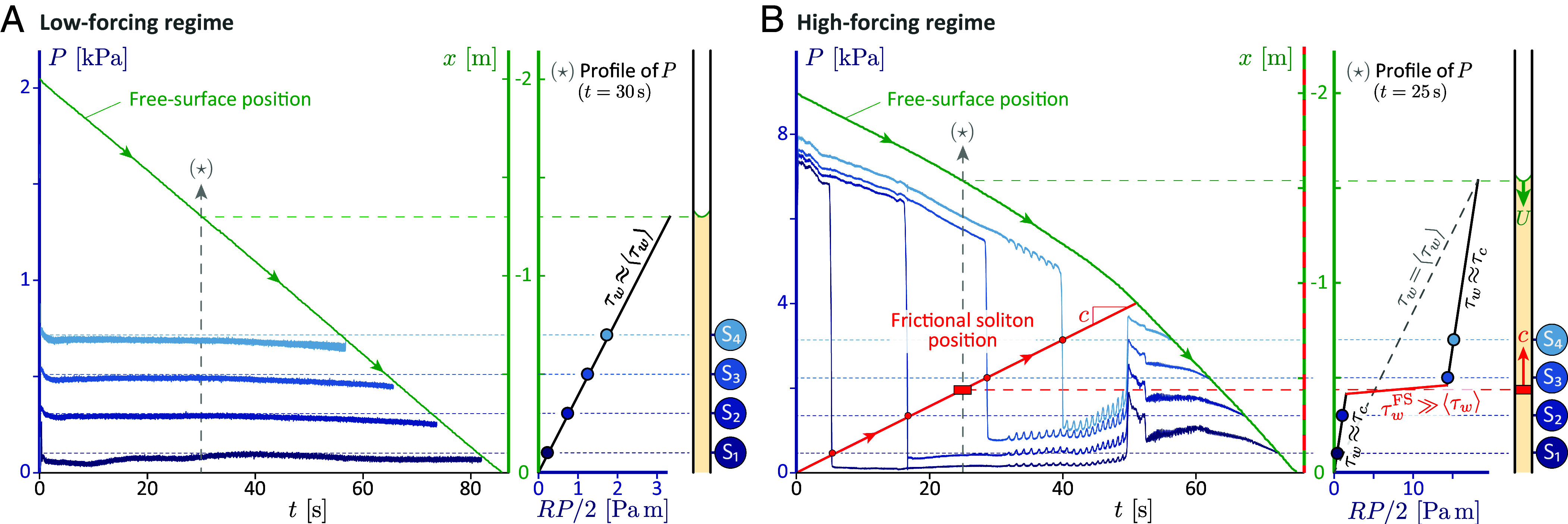
Dissipation along the pipe ( 70). (*A* and *B*, *Left*) Temporal evolution of the total flow pressure $P=P^{\prime }-\rho g\sin\theta x$ at sections $\;{\text{S}}_{1}$ to $\;{\text{S}}_{4}$ (blue curves) for $\phi =0.405>\phi_{\;\text{DST}}$ (R=5.15 mm). (*A*) Low-forcing regime ($\theta =4.9^{{}^{\circ}}$, $\langle \tau_{w}\rangle =2.6\;$Pa $<\tau_{c}$). (*B*) High-forcing regime ($\theta =22.0^{{}^{\circ}}$, $\langle \tau_{w}\rangle =11.6\;$Pa $>\tau_{c}$). Green line: free-surface position (inferred from drained suspension mass). Blue dashed lines: expected values of P for a steady uniform flow. Red line in (*B*): frictional-soliton position (red circles mark passing times at $\;{\text{S}}_{1}$ to $\;{\text{S}}_{4}$, the line is a linear fit). (*A* and *B*, *Right*) Inferred longitudinal profile of RP/2 (at fixed time). The inverse slope $\tau_{w}\equiv -R\nabla P/2$ is the local wall stress.

To obtain the evolution of the wall stress along the pipe, the bulk pressure $P^{\prime }$ (relative to ambient) is measured at several distances from the pipe outlet (see Fig. 1*A* and *M&M*). In the presence of pressure gradients and neglecting inertia, the force balance on a slice of suspension gives $\tau_{\;\text{w}}=-\left(R/2\right)\nabla \left(P^{\prime }+\rho gz\right)$, with $\tau_{w}$ the local wall stress and z=−sinθx the elevation relative to the pipe outlet. Fig. 4 presents the evolution of the reconstructed total pressure, $P=P^{\prime }+\rho gz$, which quantifies the loss between the current abscissa x and the pipe outlet, together with the time evolution of the suspension surface position −L. For low forcings ($\left\langle \tau_{w}\right\rangle <\tau_{c}\left(\phi \right)$, Fig. 4*A*), P is fixed in time at each sensor, and values from different sensors show a linear decrease along the pipe. These measurements confirm the steadiness and uniformity of the flow, and imply that the assessed local wall stress $\tau_{w}$ is uniform and equal to $\left\langle \tau_{w}\right\rangle$ (see profile of P at 30 s).

For high forcings, ($\left\langle \tau_{w}\right\rangle >\tau_{c}\left(\phi \right)$, Fig. 4*B*), the frictional soliton changes the evolution of the total pressure P qualitatively. Let us consider, first, the temporal evolution for a fixed position; that of the most downstream sensor $\;{\text{S}}_{1}$. At flow start (t=0), P is much higher than expected for a uniform resistance (≈0.5 kPa, dark blue dashed line), which indicates a large overdissipation downstream. At t≈5s, the pressure P suddenly drops to a fraction of the uniform-flow expectation (dark blue dashed line), signaling that the overdissipation has moved upstream of sensor $\;{\text{S}}_{1}$ within a very short time ($t_{\;\text{drop}}\approx 0.4$ s). Subsequently, P remains essentially constant for a long period (t≲44 s), reflecting steady losses downstream. A similar signal is obtained at sensors $\;{\text{S}}_{2}$, $\;{\text{S}}_{3}$, and $\;{\text{S}}_{4}$, albeit for the delay before pressure drop, which increases linearly with sensor distance to the pipe outlet. These sudden pressure drops are found to be exactly synchronous with the passage of the frictional soliton, as inferred from the passing time and velocity measured by near-wall flow observations between $\;{\text{S}}_{2}$ and $S_{3}$. This reveals that the frictional soliton is the locus of a superdissipation, since local (bulk) pressure drop directly reflects local dissipation (given the constant flow rate and negligible inertia). Wall stress is highly increased within a short length $\ell_{\mathrm{drop}}\equiv ct_{\mathrm{drop}}\approx 1.4R$, of order the soliton length ℓ≈3R identified from near-wall velocimetry (Fig. 3*C*). The typical magnitude of the local wall stress can be inferred from the pressure drop across the soliton, $\Delta P_{\mathrm{drop}}∼5\;$kPa, and the length $\ell_{\mathrm{drop}}$ according to $\tau_{w}∼R\Delta P_{\mathrm{drop}}/2\ell_{\mathrm{drop}}∼2\;$kPa, i.e., $\tau_{w}∼10^{2}\left\langle \tau_{w}\right\rangle$. Conversely, upstream and downstream, wall stress is uniform and steady. Measurements reveal a constant substress $\tau_{w}\approx 3.3\;$Pa ($<\langle \tau_{w}\rangle =11.6\;$Pa), which actually compares with the critical shear stress of the suspension $\tau_{c}\approx 4.0$ Pa (see profile of P at 25 s).

Additionally, the extrapolation of the soliton trajectory intersects x=0 at t=0, indicating that the soliton nucleates at the pipe outlet when flow starts (see Fig. 4*B*, *Left* and *M&M*). Crucially, when the soliton reaches the upper surface of the suspension (intersect of red and green trajectories in Fig. 4*B*, *Left*), the total pressures of the four sensors step back, synchronously, to a large value, signaling the inception of a new soliton at the pipe outlet, at the instant the first one extinguishes. This indicates that a single soliton is actually maintained to accommodate the high forcing. Last, a slight increase in the flow rate, by a typical factor 2, is observed as the second soliton replaces the first one, which suggests that the passage of the first soliton somehow modifies the distribution or structure of the suspension grains (as a reference, no change in flow rate is observed for the same drained mass in the low forcing regime, Fig. 4*A*).

Altogether, these measurements highlight that flow rate saturation at high forcings is the consequence of the inception and self-preserving upstream propagation of a single, localized, and superdissipative flow structure: a frictional soliton. The localization of high stresses in the soliton actually allows the rest of the suspension, both upstream and downstream, to sustain only a mild stress $∼\;\;\tau_{c}$, compatible with the frictionless state, hence, a laminar flow.

### Two-Flow-Phase Model of the High-Forcing Regime.

The observations reported above allow to envision the high-forcing regime as the coexistence of two flow phases, providing guides for establishing quantitative local resistance laws for all flow regimes and phases. In the low-forcing regime ($\left\langle \tau_{w}\right\rangle ≲\tau_{c}\left(\phi \right)$), the steady and uniform resistance is simply $\left\langle \tau_{w}\right\rangle$. In the high-forcing regime ($\left\langle \tau_{w}\right\rangle ≳\tau_{c}\left(\phi \right)$), the resistance of the laminar phase is close to the critical stress $\tau_{c}\left(\phi \right)$, whereas the frictional-soliton phase carries the global load $2\pi RL\langle \tau_{w}\rangle$ minus the part ≈2$\pi R\left(L-\ell \right)\tau_{c}\left(\phi \right)$ taken by the laminar phase. This can be summarized with the following laws, valid at resolution down to the cross-sectional scale R:

[1] $$\begin{array}{ll} \text{Low-forcing regime}\;\;(\langle \tau_{w}\rangle <\tau_{c}(\phi )) & \\ \;\tau_{w}=\langle \tau_{w}\rangle , & \text{everywhere}. \end{array}$$

[2] $$\begin{array}{l} \text{High-forcing}\;\text{regime}\;(\langle \tau_{w}\rangle >\tau_{c}(\phi ))\\ \;\tau_{w}\approx \left|\begin{array}{l} \tau_{c}(\phi ),\;\;\;\;\;\;\;\;\;\;\;\;\;\;\;\;\;\;\;\;\;\;\;\;\;\;\;\;\;\;\;\;\;\;\;\;\;\;\;\;\;\;\;\;\;\;\;\;\text{in}\;\text{the}\;\text{laminar}\;\text{phase},\\ \tau_{c}(\phi )+\frac{L}{l}(\langle \tau_{w}\rangle -\tau_{c}(\phi )),\;\;\;\text{in}\;\text{the}\;\text{soliton}\;\text{phase}\text{.} \end{array}\right. \end{array}$$

**Fig. 5.**
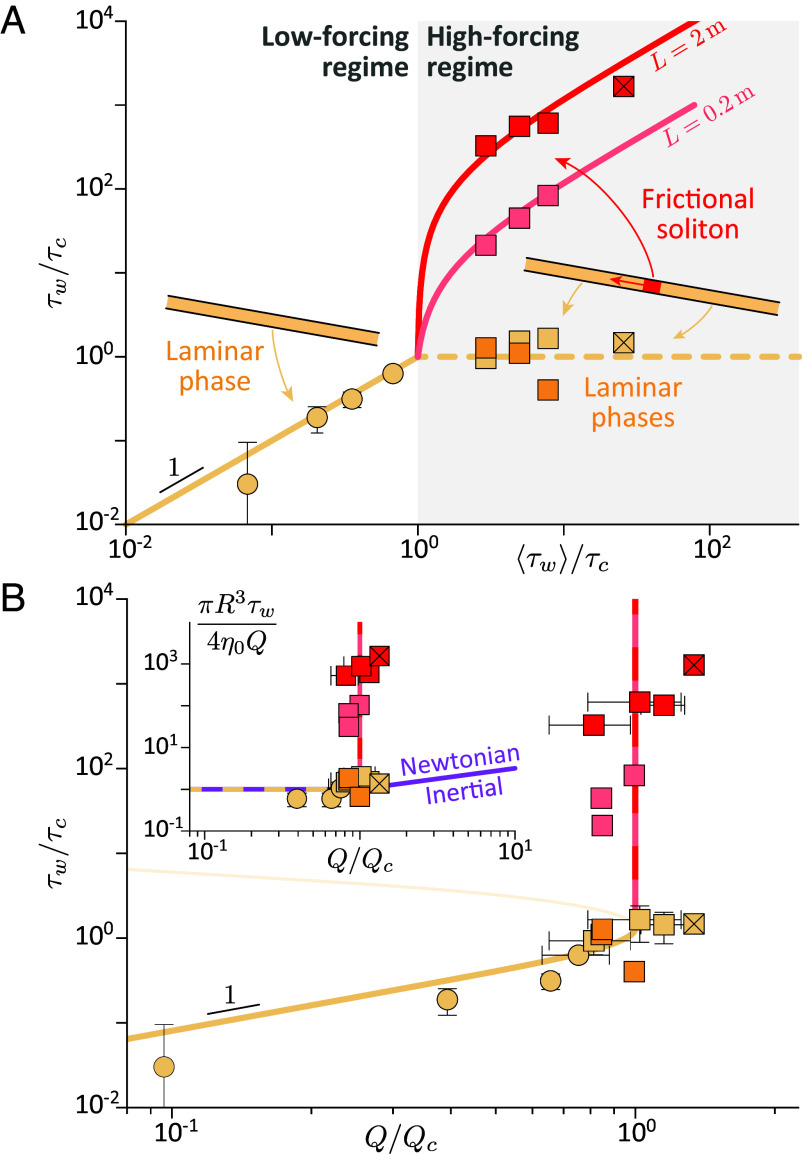
Local resistance law of the laminar and soliton phases ( 70) (ϕ=0.405). (*A*) Local wall stress vs mean applied wall stress (both scaled by the critical shear stress $\tau_{c}$ measured in Fig. 1*B*). Yellow lines: Eq. **1** (solid) and laminar branch of Eq. **2** (dashed). Red and pink lines: frictional-soliton branch of Eq. **2**, for different initial flow length (L= 0.2 and 2 m). (*B*) Local wall stress vs scaled flow rate ($Q_{c}\equiv \pi R^{3}\tau_{c}/4\eta_{0}$, with values of $\eta_{0}$ and $\tau_{c}$ obtained in Fig. 1*B*). Yellow line: base-state expectation from the Wyart–Cates rheological law. Red-pink dashed line: Eq. **4**. (*A* and *B*) Symbol color refers to L=2m (red, yellow) and L=0.2m (pink, orange). Symbol shape indicates laminar phase only (○), or laminar phase + frictional soliton (□; ⊠ indicates $\left\langle \tau_{w}\right\rangle$ is imposed by gravity + air pressure difference, see text; the wall stress in the soliton is obtained from the pressure drop across the pipe portion containing the soliton and by subtracting the laminar contribution over the length $\;{\text{S}}_{\;\text{i}}\;{\text{S}}_{\;\text{i+1}}-\ell$; vertical error bars indicate the SD between measurements over $\;{\text{S}}_{1}\;{\text{S}}_{2}$, $\;{\text{S}}_{2}\;{\text{S}}_{3}$, and $\;{\text{S}}_{3}\;{\text{S}}_{4}$). (*Inset*) Same data nondimensionalized by the laminar stress $\frac{4\eta_{0}Q}{\pi R^{3}}$ highlighting the functional difference between the frictional-soliton bifurcation (symbols, yellow and red-pink lines, Eqs. **1** and **2** with $Q_{c}=\pi R^{3}\tau_{c}\left(\phi \right)/4\eta_{0}$) and the inertial transition of a Newtonian liquid [purple line, $\frac{\pi R^{3}\tau_{w}}{4\eta_{0}Q}\approx {\left(\frac{Q}{Q_{c}}\right)}^{1/4}$ with $Q_{c}\approx {\left(\frac{64}{0.3164}\right)}^{4/3}\frac{\pi \eta_{0}R}{\rho }$ (9)].

These laws can be tested against experiments. Fig. 5*A* presents systematic measurements of the local wall stress, $\tau_{w}\equiv -R\nabla P/2$, as inferred from the pressure measurements along the pipe (see Fig. 5 caption). At low forcings ($\left\langle \tau_{w}\right\rangle <\tau_{c}\left(\phi \right)$), the local wall stress $\tau_{w}$ matches the mean imposed stress $\left\langle \tau_{w}\right\rangle$ in agreement with Eq. **1**. For high forcings ($\left\langle \tau_{w}\right\rangle >\tau_{c}\left(\phi \right)$), the local wall stress strongly depends on the flow phase. In the laminar phase (yellow and orange squares) the wall stress is close to the critical stress ($\tau_{w}\approx \tau_{c}\left(\phi \right)$), independently of the average forcing $\left\langle \tau_{w}\right\rangle$ and of the flow length L, consistently with Eq. **2**. By contrast, the local wall stress measured in the soliton phase (red and pink squares) is much larger and depends on both quantities. For a fixed flow length L, it increases linearly with the excess forcing $\left\langle \tau_{w}\right\rangle -\tau_{c}$, as anticipated by Eq. **2**. The agreement is not only observed up to the largest gravitational forcing $\left\langle \tau_{w}\right\rangle =\rho gR/2\approx 8\tau_{c}$ obtained for a vertical pipe, but also for a higher forcing $\left\langle \tau_{w}\right\rangle \approx 26\tau_{c}$ (stroked red square in Fig. 5*A*) obtained by imposing an additional pressure difference between the top and bottom of the pipe, using pressurized air. The resistance also follows the linear trend in the flow length L of Eq. **2**, as verified by using a ten times shorter pipe (pink squares in Fig. 5*A*).

Importantly, if the local resistance laws give information on the stress with a resolution down to the pipe radius—which is useful to address the load and fatigue on the pipe structure down to this scale—they also embed the global resistance law of the flow. Indeed, the pipe always contains a laminar phase, with a wall stress following $\tau_{w}\approx \frac{4\eta_{0}}{\pi R^{3}}Q$, up to the critical flow rate $Q_{c}\equiv \frac{\pi R^{3}}{4\eta_{0}}\tau_{c}\left(\phi \right)$ obtained at $\left\langle \tau_{w}\right\rangle \approx \tau_{c}\left(\phi \right)$. Integrating Eqs. **1** and **2** along the pipe thus yields:

[3] $$\begin{array}{l} \text{Low-forcing}\;\text{regime}\\ \;\left(\langle \tau_{w}\rangle <\tau_{c}(\phi )\right) \end{array}\;\;\;\;\;\;\;\;Q\approx \frac{\pi R^{3}}{4\eta_{0}}\langle \tau_{w}\rangle ,$$

[4] $$\begin{array}{l} \text{High-forcing}\;\text{regime}\\ \left(\langle \tau_{w}\rangle >\tau_{c}(\phi )\right) \end{array}\;\;\;\;\;Q\approx \frac{\pi R^{3}}{4\eta_{0}}\tau_{c}(\phi )\equiv Q_{c}(\phi ,R),$$

which is the global flow curve discussed in Fig. 2, with a linear increase at low-forcing and a saturation at high-forcing. Notably, Eq. **3** is experimentally verified for varied pipe radii (R≈3, 5, and 17 mm, see *SI Appendix*, SI.6). Therefore, flow saturates at highly different Reynolds numbers, including values much below 1 ($\rho Q/\pi \eta_{0}R\approx 0.04\;-\;7$), which indicates that flow saturation is not of inertial origin but stems from the specific rheological response of the suspension to the flow.

Last, it must be realized that the local resistance law of the soliton phase (Eq. **2**) is of a very peculiar kind and actually reveals a singularly steep rheology. This is highlighted in Fig. 5*B*, by presenting the evolution of the local wall stress $\tau_{w}$ with the pipe flow rate Q. At low flow rates ($Q<Q_{c}$), the flow is in the laminar regime and local stresses follow the global forcing, as already discussed in Figs. 2*C* and 5*A*. However, close to $Q_{c}$, a frictional soliton forms and the wall stress in the soliton shows a very steep increase (red-pink dashed line), which reflects that the frictional soliton actually behaves as a sharp flow rate limiter: it accommodates the global overload with almost no change in the flow rate it sets.

### Universality of the Frictional Soliton.

**Fig. 6.**
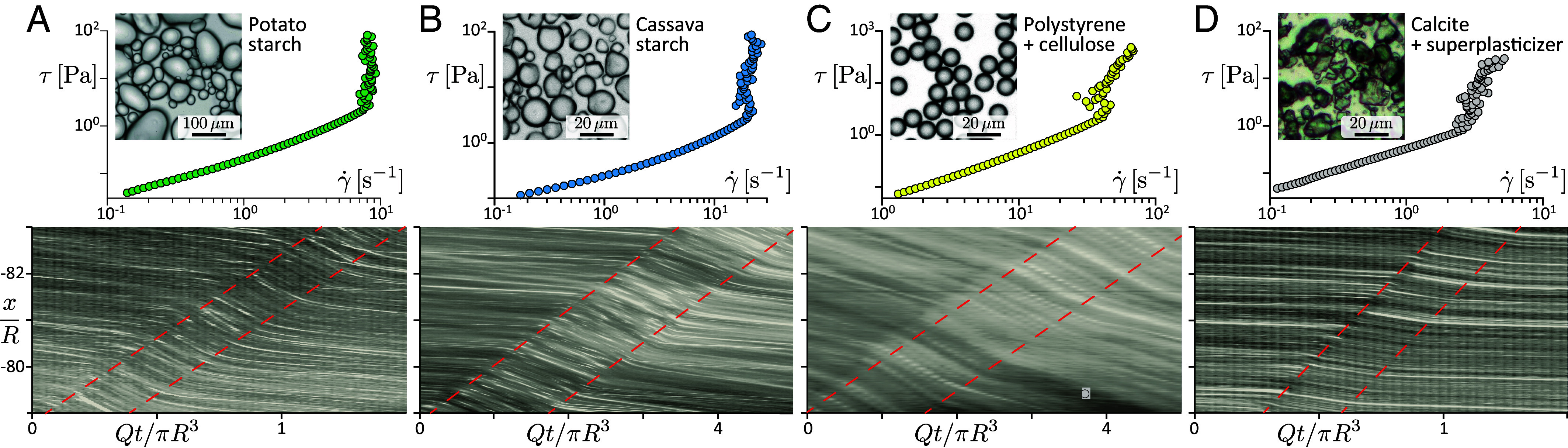
Universality of the frictional soliton phenomenology. *Top*: rheograms for four different aqueous shear-thickening suspensions (70). *Bottom*: Spatiotemporal images of the near-wall flow at large forcing highlighting the same frictional-soliton phenomenology (the origin of t is arbitrary, here). (*A*) Potato-starch, (*B*) Cassava-starch, (*C*) Polystyrene spheres coated with cellulose, (*D*) Calcite grains (CaCO_3_) + “superplasticizer” additive (polycarboxylate ether). ϕ=0.42, 0.448, 0.595, and 0.543, and $\langle \tau_{w}\rangle =4.8$, 7.7, 138.5, and 41.5 Pa, respectively, see *M*&*M* and *SI Appendix*, SI.8.

All the observations and measurements discussed so far, concern suspensions of cornstarch. To probe the general relevance of the phenomenology reported above, we extend experiments to four other types of shear-thickening suspensions (Fig. 6) having different applicative context, particle shape, size polydispersity or composition: (*A* and *B*) potato and cassava starch grains, having a presumably similar biological polymeric-induced repulsion mechanism as cornstarch grains, but a more rounded shape and broader polydispersity, (*C*) highly monodisperse polystyrene spheres, coated with repulsive cellulosic polymers, and (*D*) calcite particles (CaCO_3_)—a model fine granulometry component of high-performance concretes—which have a crystalline shape with sharp edges and are stabilized with an industrial admixture (polycarboxylate ether superplasticizer, kindly supplied by CHRYSO) aimed at improving flowability at high solid fractions. As shown in Fig. 6 (*Top*), dense aqueous suspensions of each of these particles present a similar discontinuously shear-thickening rheology, as cornstarch particles: a close to Newtonian behavior at low stress and a large and steep shear-thickening above a critical shear stress (see rheological characterization in *M&M* and *SI Appendix*, SI.8).

In all four cases, we observe a similar saturation of the pipe flow rate at large forcing, at a value approximately matching the expected saturation $Q_{c}\equiv \left(\pi R^{3}/4\eta_{0}\right)\tau_{c}$ inferred from rheological characterization ($0.8\leq Q/Q_{c}\leq 4.6$, see *SI Appendix*, SI.8). The saturation is also found to result from the inception of a frictional soliton (Fig. 6, *Bottom*), whose length compares with the pipe diameter (0.9≤l/2R≤1.5), and velocity remains of the order of the mean flow velocity (0.8≤c/U≤6.2, the larger values being obtained with particles A and D). Last, near-wall velocimetry ($2\leq U_{w}^{\mathrm{FS}}/U_{w}\leq 8.3$) recovers that the cross-sectional velocity profile changes qualitatively from Poiseuille-like to plug-like flow within the soliton. These observations confirm that the phenomenology associated to the frictional soliton is not specific to cornstarch, but in fact relevant for a broad class of shear-thickening suspensions.

## Discussion

The set of observations reported above draw a very unconventional picture of low-Reynolds pipe flows, in the particular case of shear-thickening suspensions. They reveal remarkable behaviors, both from a global perspective and in the structuration of the flow at the scale of the pipe radius R, which are controlled by the mean applied wall stress relative to the discontinuous shear-thickening onset $\tilde{\tau }\equiv \left\langle \tau_{w}\right\rangle /\tau_{c}\left(\phi \right)=-R\nabla P/2\tau_{c}\left(\phi \right)$. For highly concentrated suspensions ($\phi >\phi_{\;\text{DST}}$), a bifurcation of the flow is observed for $\tilde{\tau }≳1$: a second flow phase, coined frictional soliton, nucleates and coexists with the steady, laminar, Poiseuille-like flow obtained at low applied stress. The frictional soliton is a longitudinally localized, upstream propagative, shape-preserving flow structure, which spans the pipe cross-section. It is, above all, a superdissipative structure, concentrating most of the dissipation over a flow length of order R. Its steep (local) resistance law, summarized by Eq. **2** and valid at the pipe radius scale, actually determines the singularly steep resistance law at the whole pipe scale (Eqs. **3** and **4**), i.e., a Poiseuille law, for low applied stress, continued by a saturation of the flow rate, at higher stress, at a value $\approx \;\;Q_{c}\equiv \pi R^{3}\tau_{c}\left(\phi \right)/4\eta_{0}$ set by $\tau_{c}\left(\phi \right)$ and the low-stress (frictionless) viscosity $\eta_{0}$.

Various aspects of this remarkable phenomenology require comments. First, it does not depend on flow inertia. Regarding the inception of the frictional soliton, the same onset stress ($\tilde{\tau }=1$) is observed for a broad range of Reynolds numbers ($Re=\rho Q/\pi \eta_{0}R$$∼10^{-2}\;-\;10$), as R is varied. Regarding the self-sustained propagation, the local effective viscosity in the soliton $∼\;\;\pi R^{3}\tau_{w}^{\mathrm{FS}}/4Q_{c}$, is larger than the laminar phase viscosity $\eta_{0}$ by a factor $∼\;\;\tau_{w}^{\mathrm{FS}}/\tau_{c}\left(\phi \right)∼L/\ell$, see Eq. **2**. Hence, the relevant local Reynolds number $∼\;\;\left(\ell /L\right)Re∼10^{-4}\;-\;10^{-1}$ is much below 1, which confirms the subdominant role of inertia in the soliton dynamics.

Second, the emergence of a localized propagative flow phase (the frictional soliton) coexisting with a laminar phase, is reminiscent of other solitary waves or intermittent flow structures so far identified in pipe flows at high forcings, such as turbulent puffs and slugs in the inertial transition regime (4, 7, 8, 10), density/concentration waves in compressible or incompressible two-phase media (50–53), or bulging waves in elastically or viscously compliant pipes (54–56). However, the frictional soliton is distinct in nature from these previously reported examples. For instance, inertial puffs are memoryless structures, which stochastically nucleate, decay, and coexist (6, 7), without affecting drastically the global resistance or flow rate, because the local resistance law of the puff/slug phases is not steep enough (9) (*Inset* of Fig. 5*B*). In contrast, frictional solitons are characterized by their deterministic uniqueness—in the range of parameter investigated, one and only one soliton actually emerges and propagates above $\tau_{c}\left(\phi \right)$—and their singularly steep resistance law, Eq. **2**. This law and the low bulk compressibility of the suspension (liquid+grains) imply that the nucleation of a frictional soliton affects the resistance of the whole pipe flow within milliseconds ($∼\;\;\;L/c_{\mathrm{sound}}$, with $c_{\mathrm{sound}}∼1\;$ km/s the sound speed), and acts as a particularly efficient flow limiter, accommodating large variations in the total applied load with minimal change in the flow rate. They also allow the uniqueness of the soliton, since the resulting saturation of the flow rate at $Q_{c}$ maintains the coexisting flow phase in a frictionless, hence laminar, state. Note, however, that a different phenomenology might emerge for sufficiently slender pipes or high applied stresses, since the stress in the soliton $∼\;\;\left(L/R\right)\left\langle \tau_{w}\right\rangle$ could deform the particles or trigger cavitation.

The phenomenology of the frictional soliton also differs, qualitatively, from the mechanisms proposed so far for other unsteady shear-thickening flows. At high stress, these flows feature large stress and velocity fluctuations (29, 36, 57), but these are small scale, intermittent or chaotic structures. Coherent propagative structures, such as jamming fronts, have also been reported in shear flows, but these are intrinsically inertial fronts which propagate transversely to the flow with a large velocity depending strongly on volume fraction (35, 41–44). Stress and concentration bands traveling either parallel (36, 37) or perpendicular (30, 31) to the flow direction have also been observed and modeled, but none of them have been shown to develop upstream propagative solitary waves. Specific noninertial surface waves have also been reported and modeled (33, 34, 58), but the mechanism, involving the free surface, does not apply to wall-bounded flows. Last, upstream propagative density waves have been observed for flows in capillaries (52, 53), but these are not mutually exclusive and develop only in capillaries with diameter of a few particle sizes. Perhaps the most similar phenomenology to the frictional soliton has been observed in a plate-plate Couette flow, with jammed structures propagating both upstream and downstream at speeds close to the forcing velocity (36, 38, 39, 59). No mechanism has been proposed so far, but pore-scale measurements of the relative velocity between the particles and the suspending liquid suggest that these structures involve strongly localized dilation of the suspension (39), which is also consistent with the bubble expansion observed in the frictional soliton (Fig. 3*D*).

**Fig. 7.**
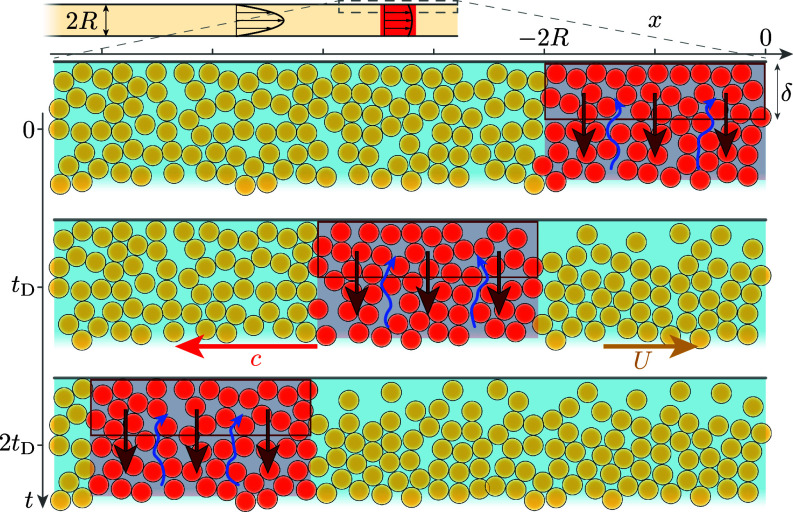
Minimal mechanism for the frictional-soliton propagation. As the soliton passes a slab of suspension: (*i*) the shear stress builds up above $\tau_{c}$ and the suspension jams over a length scale set by the pipe diameter 2R, (*ii*) the steady flow rate forces the jammed suspension, which dilates at the wall, over a layer of thickness δ and a time scale $t_{\;\text{D}}∼\gamma_{0}/\dot{\gamma }∼\gamma_{0}R/U$, (*iii*) because of the particle-depleted layer, the local shear stress relaxes below $\tau_{c}$, which builds up the shear stress immediately upstream. The soliton thus propagates upstream at a velocity $c∼\ell /t_{\;\text{D}}∼2U/\gamma_{0}$, of the order of the mean flow velocity U.

These latter observations suggest that particle dilation and the coupling with the suspending liquid are essential ingredients to explain the propagation and the high dissipation in the frictional soliton. We propose the following minimal mechanism based on the observed length of the soliton and its formation at the pipe outlet. As the soliton passes a slab of suspension, the shear stress close to the wall builds up above the onset jamming stress, of order $\tau_{c}\left(\phi \right)$, beyond which the suspension must dilate to flow. This Reynolds-like dilation of the particle phase (60), red arrows in Fig. 7] lowers the particle volume fraction in a thin layer of suspension close to the wall and eventually the drag of the suspension slab, until the local shear stress decreases below the critical stress $\tau_{c}\left(\phi \right)$ and the suspension slab returns to a laminar flow. The dilation occurs over a typical strain $\gamma_{0}∼O\left(1\right)$ independent of ϕ (61, 62), i.e., over a typical time scale $t_{D}∼\gamma_{0}/\dot{\gamma }∼\gamma_{0}R/U$, since the near-wall shear rate in the soliton is of order U/R (*SI Appendix*, SI.4). Given the formation of the soliton at the pipe outlet and its length ℓ∼2R, this explains why the soliton propagates upstream and with a velocity $c∼\ell /t_{D}∼2U/\gamma_{0}$ of the order of the mean flow velocity.

This mechanism also explains how the stress $\tau_{w}$ in the soliton accommodates the possibly large excess load imposed at the pipe scale. The dilation involves a flow of the suspending liquid across the pores (blue arrows), which results in a large negative pore pressure, i.e., a large positive particle stress, whose magnitude is given by a Darcy–Reynolds scaling $\tau_{w}∼\left(\eta_{l}/\kappa \right)\left(\Delta \phi /\gamma_{0}\right)\delta^{2}U/R$ (62, 63), with $\eta_{l}$ the suspending liquid viscosity, $\kappa ∼10^{-3}d^{2}$ the permeability of the particle phase, d the particle diameter, $\Delta \phi ∼10^{-2}$ the distance to jamming, and δ the thickness of the dilation layer (Fig. 7). A dilation layer of the order of the pipe radius (δ∼R∼300d) is enough to build a wall stress $\tau_{w}∼10{\left(\delta /d\right)}^{2}\eta_{l}U/R∼10^{4}\eta_{0}U/R$, orders of magnitude larger than the laminar phase shear stress $∼\;\;\eta_{0}U/R$, providing room to accommodate a high external load. This minimal dilation mechanism captures the main features of the soliton phenomenology. It also agrees qualitatively with the observation of a slight, but systematic, increase in the flow rate as the second generation of frictional soliton propagates in the pipe (the particle-depleted layer is expected to shift the critical flow rate toward larger values). However, the nucleation mechanism and the selection of the soliton length remain to be elucidated. Further modeling and additional experimental inputs, in particular about the evolution of the concentration and velocity profiles, are also required to understand how the flow sets the near-wall shear rate and the thickness of the dilation layer δ.

Last, the singular steepness of the global resistance law and the frictional soliton are robustly observed with various particle systems (different natural starch grains, polymer-grafted plastic beads, calcite powder stabilized with a superplasticizer), which supports their universality for pipe flows of shear-thickening suspensions and their potential relevance to improve pumping in industrial applications. For instance, in civil engineering, superplasticizers, which are commonly added with fine materials (fly ash, silica fume) to enhance concrete flowability, make the concrete shear-thickening (64–68). This concrete is often pumped through long ($L/R≳10^{4}$) and wide pipes ($R/d≳10^{3}$) at high mean wall stress (reaching $10^{2}\;-\;10^{3}\;\tau_{c}$), and further study will have to determine whether the frictional-soliton phenomenology extends to such large forcings.

## Materials and Methods

### Setup.

The pipe is a 2m-long, transparent, smooth, PMMA tube, with inner radius R≃3, 5.15 or 17 mm. The scale has a precision of 0.01 g and a response time of 0.2 s. The four pressure gauges $S_{1}$ to $S_{4}$ are located 0.1, 0.3, 0.5, and 0.7 m from the pipe outlet, respectively. Different gauges (with ranges from 3.7 to 100 kPa, precisions from 0.1 to 1 kPa, and a response time of 1 ms) are used for the different forcings. The near-wall flow is imaged between $S_{2}$ and $S_{3}$, under laser diode illumination (2 W, 532 nm), with a camera (JAI, 4,096 × 3,072 pixels) at a spatial resolution of 10μm/pixel and acquisition rate up to 300 Hz. The fluorescent tracing particles are 20μm, spherical, PMMA particles doped with rhodamine 6G (69). To prevent image distortion by the curved pipe wall, the pipe is surrounded with a rectangular chamber (40×25×25 mm) filled with a liquid (Triton X-100) of same refractive index as the pipe wall.

### Materials.

The shear-thickening suspensions consist mainly of commercial cornstarch grains (Maisita^®^, $\rho_{p}=1,550\;$ kg/m^3^) mixed with microfiltered water ($\rho_{w}=997$ kg/m^3^). The particle volume fraction ϕ of the suspensions is computed from the weight and density of the dry particles and liquid. The suspension density is given by $\rho =\rho_{p}\phi +\rho_{w}\left(1-\phi \right)$. Prior to use, the starch is systematically desiccated with the same protocol (a few days at 60 °C in an oven) to avoid shifts in dry density due to air moisture variations. The potato starch grains, cassava starch grains and polystyrene spheres are commercial (respectively, Roquette^®^ with $\rho_{p}=1,500\;$ kg/m^3^, New Land^®^ with $\rho_{p}=1,550\;$ kg/m^3^, and TS by Dynoseeds^®^, with diameter 10.1±0.3μm and $\rho_{p}=1,050\;$ kg/m^3^). The calcite particles (Omya^®^, $\rho_{p}=2,700\;$ kg/m^3^) are sieved with a 30μm mesh. The industrial superplasticizer (polycarboxylate ether, kindly supplied by Chryso^®^) is added to the suspension at 3 wt‰.

### Rheological Characterization.

The rheograms in Figs. 1*B* and 6*A*–*C* are obtained using rough, coaxial cylinders (height of 38.7 mm, inner and outer radii of 13.55 and 15.63 mm, respectively). The flow curve are obtained as the average over three logarithmically increasing torque ramps, after a preshear. To limit migration effects above the shear-thickening onset, the suspension is floated on a heavier, nonmiscible and low-viscosity oil (perfluorotributylamine, Fluorinert FC-43). Because calcite (much denser than water and Fluorinert) settles rapidly, the Newtonian-effective rheogram in Fig. 6*D* is obtained from the torque on a homemade helix (diameter of 35 mm, with two levels of tilted blades) rotating at a low Reynolds number (≤0.3) inside a rough cup (diameter of 39 mm), which is calibrated against a potato starch shear-thickening suspension.

### Experimental Procedure and Additional Tests.

Procedure (variations, in parenthesis, have been tested without change on the phenomenology): i) The pipe is inclined at $\theta =7^{{}^{\circ}}$, below the shear-thickening threshold, and slowly filled with the freshly mixed suspension from one end (or the other), while it is continuously rotated to prevent sedimentation. ii) The pipe outlet is obturated and tilted to the desired flow inclination θ, with the outlet located ∼2R above (or beneath) the surface of a water pool to reduce (cancel) stress in the extruded suspension jet. iii) The plug is removed to start the flow. Additional tests: i) The frictional soliton can be incepted after flow start, either by increasing the inclination above the critical only after flow initiated, or by removing a rod placed across the pipe to obstruct the flow. ii) Use of a roughened pipe (obtained by gluing two sand-blasted half-pipes) yields the same saturation flow rate ($Q/Q_{c}\approx 0.5$ to 1), soliton length (ℓ/2R≈0.3 to 1) and soliton velocity (c/U≈1.6 to 2.5).

## Supplementary Material

Appendix 01 (PDF)

Movie S1. show the flow in the low-forcing regime and in the high-forcing regime, respectively (*φ* = 0.405). low-forcing regime (Fig 3A, θ = 4.9°, i.e., ⟨*τ_w_*⟩ = 2.6 Pa and ⟨*τ_w_⟩/τ_c_* ≈ 0.7, Q ≈ 1.36 ml/s).

Movie S2. show the flow in the low-forcing regime and in the high-forcing regime, respectively (*φ* = 0.405). high-forcing regime (Fig 3B, θ = 22.0°, i.e., ⟨*τ_w_*⟩ = 11.6 Pa and ⟨*τ_w_⟩/τ_c_* ≈ 2.9, Q ≈ 1.24 ml/s).

Movie S3. shows the growth of small air bubbles, fortuitously transported by the suspension, as the frictional soliton passes (Fig 3D, φ = 0.39, θ = 42.0°, i.e., ⟨*τ_w_*⟩ = 20.5 Pa and ⟨*τ_w_⟩/τ_c_* ≈ 3.7).

## Data Availability

Experimental data have been deposited in Zenodo (http://doi.org/10.5281/zenodo.10287701) (70).
