# Supplementary material for: A frictional soliton controls the resistance law of shear-thickening suspensions in pipes
Source: Proc Natl Acad Sci U S A. 2024 Apr 16;121(17):e2321581121. doi: 10.1073/pnas.2321581121 (PMC11046699; doi:10.1073/pnas.2321581121)
Supplement: Supplementary file 1 — Appendix 01 (PDF) [file pnas.2321581121.sapp.pdf]

Supplementary Information for 'A frictional soliton controls the resistance law of shear-thickening suspensions in pipes'

Alexis Bougouin[a], Bloen Metzger[a,1], Yoel Forterre[a], Pascal Boustingorry[b], and Henri Lhuissier[a]

[a]Aix Marseille Univ, CNRS, IUSTI, Marseille, France; [b]CHRYSO, 7 rue de l'Europe, F-45300 Sermaises, France

Contents of this file

SI.1. Description of the experimental movies
SI.2. Preliminary experiments with a reservoir at the pipe inlet
SI.3. Laminar base-state flow expected for a Wyart-Cates rheology
SI.4. Estimation of the cross-sectional profile of velocity in the frictional soliton
SI.5. Contribution of diffusion to the transient growth of microscopic gas bubble in the frictional soliton
SI.6. Independence of flow rate saturation on the Reynolds number of the flow
SI.7. Sampling of the particle volume fraction at the pipe outlet
SI.8. Main characteristics of the effective rheology and of the frictional soliton for the different shear-thickening suspensions

SI.1. Description of the experimental movies

The movies show the near-wall flow for a cornstarch suspension. They correspond to the spatio-temporal diagram provided in Fig. 3 of the main body of the paper.

Movies 1 & **2** show the flow in the low-forcing regime and in the high-forcing regime, respectively ($\phi = 0.405$). **Movie 1:** low-forcing regime (Fig 3A, $\theta = 4.9°$, i.e., $\langle\tau_w\rangle = 2.6$ Pa and $\langle\tau_w\rangle/\tau_c \approx 0.7$, $Q \approx 1.36$ ml/s). **Movie 2:** high-forcing regime (Fig 3B, $\theta = 22.0°$, i.e., $\langle\tau_w\rangle = 11.6$ Pa and $\langle\tau_w\rangle/\tau_c \approx 2.9$, $Q \approx 1.24$ ml/s).

Movie 3 shows the growth of small air bubbles, fortuitously transported by the suspension, as the frictional soliton passes (Fig 3D, $\phi = 0.39$, $\theta = 42.0°$, i.e., $\langle\tau_w\rangle = 20.5$ Pa and $\langle\tau_w\rangle/\tau_c \approx 3.7$).

For all three movies, the pipe radius is $R = 5.15$ mm and spatial resolution is 10 µm/pixel. Lengths are indicated by scale bars. Movie 1 and 2 are displayed in real time. Movie 3 is slowed-down by a factor 10. More information about optical measurements are given in *M&M* in the main body of the paper.

SI.2. Preliminary experiments with a reservoir at the pipe inlet

Preliminary experiments have been conducted with a horizontal, smooth PMMA tube (length $L = 0.5$ m, inner radius $R = 1.6$ mm) connected to a large feed reservoir (Fig. SI.1). The flow is driven by setting a pressure difference, $\rho gH + P_{\mathrm{air}}$, between the pipe inlet and outlet, with the help of a constant air overpressure P_{air}. In the case where inertial effects are small and the localized entrance dissipation is small relative to the regular dissipation along the pipe, this corresponds to a mean applied pressure gradient along the pipe $\langle -\nabla P\rangle \equiv (\rho gH + P_{\mathrm{air}})/L$. The pressure in the pipe is measured with sensors located 0.1, 0.2, 0.3 and 0.4 m from the pipe outlet.

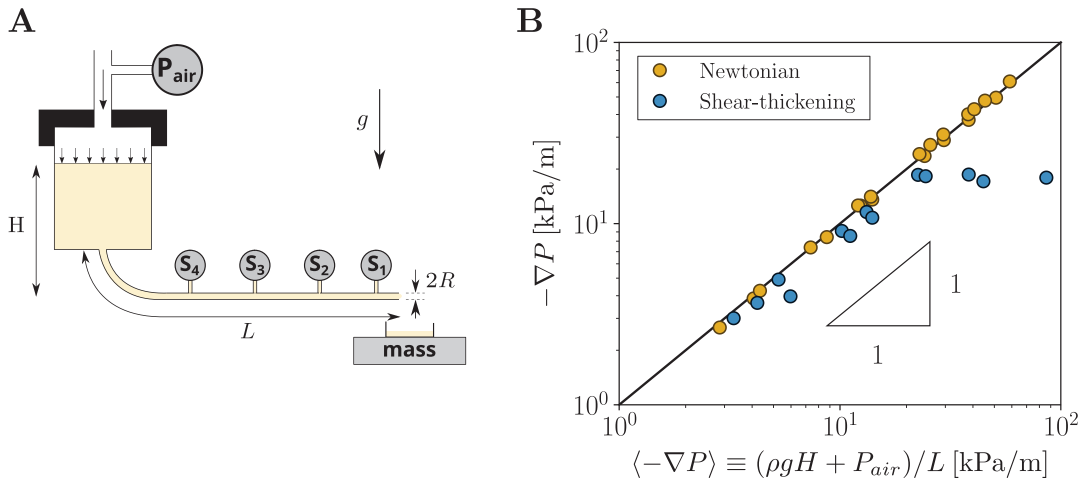

Fig. SI.1. (A) Sketch of the setup of the preliminary experiments with a feed reservoir. (B) Local pressure gradient in the pipe versus mean applied pressure gradient for a Newtonian liquid (see text) and a shear thickening suspension (10 µm polystyrene spheres in water, $\phi = 0.59 > \phi_{\mathrm{DST}} \approx 0.575$).

For experiments with a Newtonian liquid (40%w aqueous solution of PEPG (3.9 kg/mol poly(ethylene glycol-ran-propylene glycol)-monobutyl-ether by Sigma-Aldrich) with viscosity $\eta \approx 0.4$ Pa s and density $\rho \approx 1066\,\mathrm{kg/m^3}$) at low Reynolds number ($\lesssim 0.2$), the local pressure gradient $-\nabla P$, as measured in the pipe, is found to be very close to the mean pressure gradient $\langle -\nabla P\rangle \equiv (\rho g H + P_{\mathrm{air}})/L$. This agrees with the expectation, for the present case of a long pipe ($L/R \sim 300 \gg 1$), that localized entrance losses ($\sim \eta U/R$, with U the mean flow velocity) are small relative to the regular losses along the pipe ($\sim \eta UL/R^2$).

By contrast, experiments with shear-thickening suspensions reveal a mismatch between the local pressure gradient $-\nabla P$ and the mean imposed gradient $\langle -\nabla P\rangle$, at high applied pressure. For $\langle -\nabla P\rangle \gtrsim 20$ kPa/m, the gradient in the pipe actually saturates at a value $-\nabla P \sim 20$ kPa/m (blue disks in Fig. SI.1). This saturation of $-\nabla P$ is associated with a saturation of the flow rate (data not shown), which agrees with observations by (1) on a similar configuration. Importantly, our measurements show that for $\langle -\nabla P\rangle \gtrsim 20$ kPa/m the gradient is identical over each measurement portion of the pipe (S_1S_2, S_2S_3 and S_3S_4), and fixed in time. This indicates that the converging flow at the entrance of the pipe causes a large localized dissipation (presumably similar with that reported for the flow of a shear-thickening suspension through an orifice (2)), which is *fixed* at the pipe inlet and affects the whole pipe flow.

To prevent these large entrance effects and address the intrinsic flow in a pipe, we have used the drainage setup presented in the main body of the paper.

SI.3. Laminar base-state flow expected for a Wyart-Cates rheology

Model. For a steady laminar flow, driven by a uniform gravitational component $g\sin\theta$, the longitudinal velocity $u(r)$ at radial coordinate r is

$$u(r) = \int_r^R \dot\gamma(r)\,\mathrm{d}r = \int_r^R \frac{\tau(r)}{\eta(r)}\,\mathrm{d}r\,, \qquad \text{[SI.1]}$$

with $\dot\gamma(r) = -\partial u/\partial r = \tau(r)/\eta$ the local shear rate, $\tau(r) = \rho g r \sin\theta/2$ the local shear stress, ρ and η the density and effective viscosity of the flowing material, respectively, and R the pipe radius, at which a no-slip condition ($u(R) = 0$) is assumed (Fig. SI.2A). Making use of $r = 2\tau/\rho g\sin\theta$, Eq. [SI.1] can be recasted into

$$u(\tau) = \frac{2}{\rho g \sin\theta}\int_\tau^{\tau_w} \frac{\tau}{\eta(\tau)}\,\mathrm{d}\tau\,, \qquad \text{[SI.2]}$$

with $\tau_w = \langle\tau_w\rangle = \tau(r = R)$ the uniform wall stress.

The model rheological shear-thickening laws proposed by Wyart-Cates (3), assumes that the effective viscosity of the suspension depends on the magnitude of the shear stress relative to the inter-particle repulsive stress scale τ^*, according to $\eta = \eta_S(\phi_J - \phi)^{-2}$, with η_S a prefactor of order the suspending liquid viscosity, $\phi_J = (1-f)\phi_0 + f\phi_1$ the jamming volume fraction for a given stress τ, ϕ_0 and ϕ_1 the frictionless and frictional jamming volume fractions, respectively, and $f = \exp(-\tau^*/\tau)$ the stress-dependent fraction of frictional contacts between the particles in the suspension, i.e.,

$$\eta(\tau) = \eta_S\,[\phi_0 - (\phi_0 - \phi_1)\mathrm{e}^{-\tau^*/\tau} - \phi]^{-2}\,, \qquad \text{[SI.3]}$$

of which the four physical parameters (η_S, ϕ_0, ϕ_1, τ^*) must be determined from rheological measurements.

Combining Eqs. [SI.2-SI.3], the velocity profile and the flow rate, are obtained, respectively, as

$$u(\tau) = \frac{2}{\eta_S \rho g \sin\theta}\int_\tau^{\tau_w} \frac{\tau}{[\phi_0 - (\phi_0-\phi_1)\mathrm{e}^{-\tau^*/\tau} - \phi]^{-2}}\,\mathrm{d}\tau\,, \qquad Q(\tau) = \frac{2\pi R^2}{{\tau_w}^2}\int_0^{\tau_w} u(\tau)\tau\,\mathrm{d}\tau\,. \qquad \text{[SI.4]}$$

Rheological data fitting procedure. The parameters η_s, ϕ_0, ϕ_1 and τ^* for the cornstarch suspensions are obtained by fitting Eq. [SI.3] to the rheological measurements (see *M&M* in the main body of the paper for information about the measurements). First, the low stress (circles) and high stress (squares) viscosity branches, are jointly fitted with $\eta = \eta_S(\phi_0 - \phi)^{-2}$ and $\eta = \eta_S(\phi_1 - \phi)^{-2}$, respectively, to determine $\eta_S = 0.28$ mPa.s, $\phi_0 = 0.445$ and $\phi_1 = 0.385$ (Fig. SI.2B-left). This also sets the minimal volume fraction for discontinuous shear thickening $\phi_{\mathrm{DST}} = \phi_0 - 2\mathrm{e}^{-1/2}(\phi_0 - \phi_1) \approx 0.37$. Second, the repulsive stress scale τ^* is obtained by fitting the whole data set, which gives $\tau^* = 8.0$ Pa (Fig. SI.2B-right). Despite their simplicity, the Wyart-Cates rheological laws are found to fit fairly well the global trends of the rheological measurements (except for the negatively-sloped region, where measurements are not expected to reflect the rheological response of the suspension because of flow instabilities leading to large deviations from a laminar rheometric flow (4–6)). In particular, they fit fairly well the evolution of the non-frictional viscosity, $\eta_0(\phi) \equiv \eta_S(\phi_0 - \phi)^{-2}$, and of the critical shear stress, $\tau_c(\phi)$, with the particle volume fraction ϕ.

Laminar base-state velocity profile and flow rate. Fig. SI.2C, presents the laminar base-state velocity profile and flow rate (Eq. [SI.4]) using the rheological parameters fitted on the rheograms of cornstarch suspensions (Fig. SI.2B). The velocity profile, normalized by the maximal velocity $u_{\max} = u(r = 0)$, is plotted for a fixed volume fraction $\phi = 0.41 > \phi_{\mathrm{DST}}$ and relative wall stresses τ_w/τ^* ranging from 0.1 to 10. The normalized flow rate, $\eta_S Q/R^3\tau^*$, is plotted as a function of τ_w/τ^* for particle volume fractions between 0.30 and 0.44. For low wall stresses ($\tau_w \ll \tau_c \approx 0.4\tau^*$), the velocity profile is close to parabolic and the flow rate follows Hagen-Poiseuille law $Q = \frac{\pi R^3}{4\eta_0}\langle\tau_w\rangle \propto \langle\tau_w\rangle$, with $\eta_0 = \eta_S(\phi_0 - \phi)^{-2}$ the frictionless viscosity. As stress is increased much above τ_c, an increasingly large portion of the suspension next to the wall is expected to jam, and the base-state flow rate decreases, asymptotically, as $Q \sim (R^3\tau_c/\eta_0) \times (\tau_c/\tau_w)^3 \propto \tau_w^{-3}$.

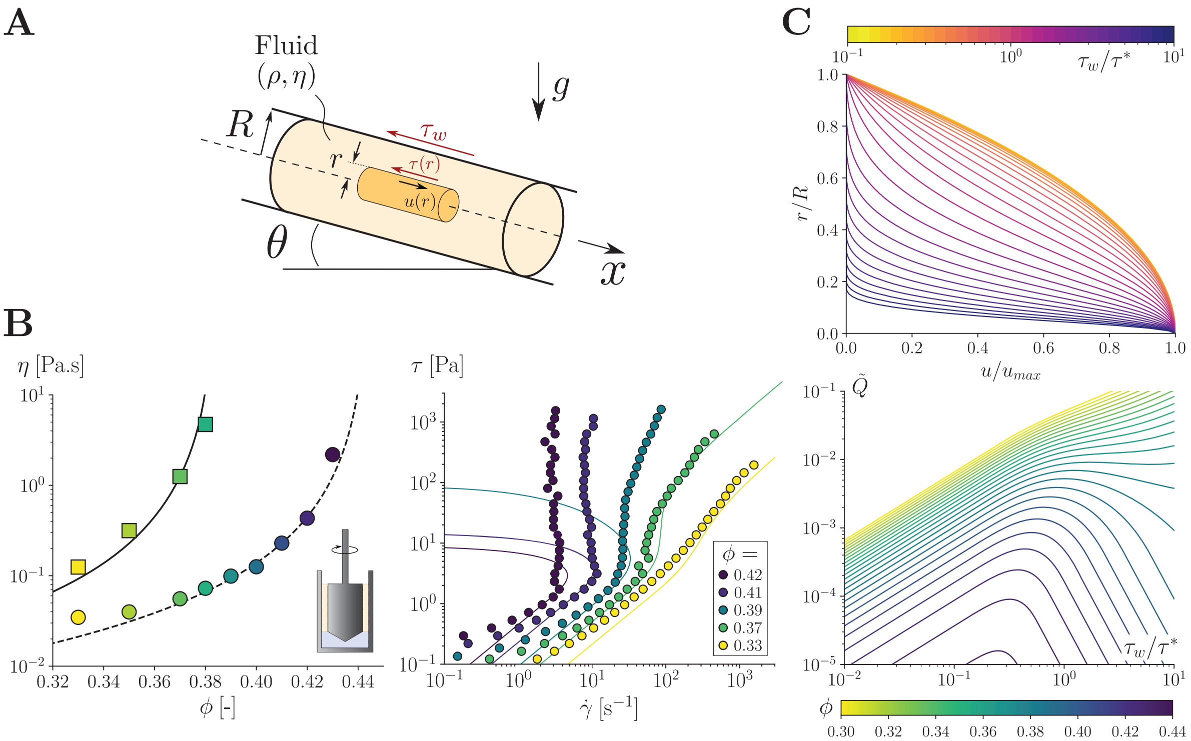

Fig. SI.2. (A) Force balance within the pipe cross-section. (B-left) Low-stress (circles) and high-stress (squares) viscosity branches. Dashed line: $\eta = \eta_S\,(\phi_0 - \phi)^{-2}$. Solid line: $\eta = \eta_S\,(\phi_1 - \phi)^{-2}$. (B-right) Shear stress τ versus shear rate $\dot{\gamma}$ for different volume fractions ϕ. Solid lines: fitted Wyart-Cates rheological laws. $\eta_S = 0.28$ mPa.s, $\phi_0 = 0.445$, $\phi_1 = 0.385$ and $\tau^* = 8.0$ Pa. (C-top) Normalized velocity profile, $u(r)/u_{\max}$ (Eq. [SI.4], with $u_{\max} = u(0)$), for different wall stress τ_w/τ^* and $\phi = 0.41$. (C-bottom) Normalized flow rate, $\tilde{Q} = \eta_s Q/R^3\tau^*$, versus τ_w/τ^*, for various ϕ. The parameters η_S, ϕ_0, ϕ_1 and τ^* are those obtained from the rheological measurements.

SI.4. Estimation of the cross-sectional profile of velocity in the frictional soliton

The opacity of the suspension restricts the observation of the flow to within a short distance λ from the wall (set by the laser penetration depth through the suspension). By calibrating the near-wall flow observations against the Poiseuille flow expected in the low-forcing regime, we estimate the typical slip velocity and the typical velocity gradient at the wall in the frictional soliton.

Two quantities are extracted from the movies: the mean flow velocity U_w within the near-wall observation depth λ, and variations of the flow velocity across the same depth (see variations in the slope of the spatio-temporal trajectories of the tracing particles in Fig. 3A-B of the main body of the paper). They are both obtained by measuring, for each of the two flow phases, the velocity component parallel to the pipe axis of 30 to 40 tracing particles randomly chosen within the observation depth.

Estimation of the near-wall observation distance. The near-wall observation distance λ is estimated from low-forcing flows, assuming a Poiseuille velocity profile. For a Poiseuille flow, the longitudinal velocity $u(r)$ follows a parabolic profile $u(r) = 2U(1 - r^2/R^2)$ relative to the radial coordinate r, with U the mean flow velocity and R the pipe radius. This means that the wall-distance $\lambda = R - r$ at which a given velocity U_w is observed follows

$$\frac{\lambda}{R} = 1 - \sqrt{1 - \frac{U_w}{2U}} \simeq \frac{U_w}{4U}. \qquad \text{[SI.5]}$$

From the velocity $U_w \approx 0.10U$ observed at low forcings ($\phi = 0.405$, $\langle\tau_w\rangle \approx 0.7\tau_c(\phi)$), one estimates the effective observation distance to the wall as $\lambda \approx 0.025R \approx 120$ µm, or $\approx$ 5-10 cornstarch grain diameters of ≈ 15 µm, given $R = 5.15$ mm.

Estimation of the cross-sectional profile of velocity in the frictional soliton. The mean near-wall velocity in the frictional soliton is $U_w^{\mathrm{FS}} \approx 0.6U$ (for $\langle\tau_w\rangle \approx 2.9\tau_c(\phi)$), with U of the mean flow velocity in the pipe, as compared to $U_w \approx 0.10U$ for the laminar phases (all measurements are performed at $\phi = 0.405$). The relative variations in velocity across the observation depth is $\Delta U_w^{\mathrm{FS}}/U \equiv \sqrt{\langle u^2\rangle - (U_w^{\mathrm{FS}})^2}/U \approx 0.077$ in the soliton, as compared to $\Delta U_w/U \equiv \sqrt{\langle u^2\rangle - U_w^2}/U \approx 0.027$ for the laminar phases (independently of the level of applied stress, as long as $\langle\tau_w\rangle < \tau_c(\phi)$). Interpreting this variation as a proxi for the near-wall velocity gradient, i.e., $-\partial u/\partial r|_{r=R} \propto \Delta U_w/\lambda$ (since $\lambda/R \approx 0.025 \ll 1$), yields $-\partial u/\partial r|_{r=R} \approx 11U/R$ in the soliton, as compared to $-\partial u/\partial r|_{r=R} = 4U/R$ for the laminar phases (assuming, again, a Poiseuille velocity profile $u(r) = 2U(1 - r^2/R^2)$ in the laminar phases).

Altogether, these measurements suggests that the cross-sectional profile of velocity in the soliton is closer to a plug flow, with a significant slip velocity ($u^{\mathrm{FS}}(r = 0) \approx U_w^{\mathrm{FS}} + \lambda\partial u/\partial r|_{r=R} \approx 0.3U$) and a velocity gradient at the wall ($-\partial u/\partial r|_{r=R} \approx 11U/R$) of the same order of magnitude, though a few times larger, than in the laminar phases ($-\partial u/\partial r|_{r=R} = 4U/R$), as schematized in Fig. 3B of the main body of the paper.

SI.5. Contribution of diffusion to the transient growth of microscopic gas bubble in the frictional soliton

Microscopic gas bubbles, which are fortuitously trapped in the suspension, are found to expand, as the soliton passes, and to collapse, immediately after. This reflects the decrease of the liquid pressure within the soliton. In the main text, the magnitude of the pressure drop is estimated by assuming that the bubble growth is essentially due to the inflation of the gas that is initially inside the bubble. This demands that diffusive transport of gas from the solution to the bubble has a negligible contribution to the growth, which is what this appendix shows.

The radius $r(t)$ of a spherical bubble, growing by mass-limited diffusion in a non-moving supersaturated liquid, follows (7)

$$r^2(t) \approx r_0^2 + 2\frac{\Delta c}{\rho_g} Dt\,, \qquad \text{[SI.6]}$$

in the limit of both long times ($t \ll R^2/D$) and low supersaturation ($\Delta c/\rho_g \ll 1$), with r_0 the initial bubble radius, ρ_g the gas density inside the bubble, Δc the gas supersaturation of the liquid relative to the bubble condition expressed in kg/m^3, D the diffusion coefficient of the gas in the liquid, and t the time since $r = r_0$. For a sudden and large pressure drop, the supersaturation is (at most) equal to the density of gas dissolved in the liquid. Therefore, the supersaturation is also (at most) equal to the saturated density $c_{\text{sat},1\,\text{atm}}$, assuming that the liquid is close to saturation upstream of the soliton, where the pressure is $P \approx 1$ atm, consistently with the observation that bubble size is not varying rapidly, there.

This, together with Eq. [SI.6], implies that the diffusive growth time is approximately:

$$t \approx \frac{1}{2}\frac{\rho_g}{c_{\text{sat},1\,\text{atm}}}\frac{r^2 - r_0^2}{D}. \qquad \text{[SI.7]}$$

The initial bubble radius is $r_0 \approx 20\,\mu\text{m}$. The maximal radius $r(t)$ is (at least) twice as large. The gas density in the bubble is $\rho_g \approx 1.2\,\text{kg/m}^3$. Considering the contribution of nitrogen and oxygen, only, because they dominate the diffusive growth in air-equilibrated water, one has $c_{\text{sat},1\,\text{atm}} \approx 17\,\text{g/m}^3$ and $D \approx 2.2 \times 10^{-9}\,\text{m}^2/\text{s}$.

Evaluating Eq. [SI.7] yields $t \approx 19$ s (at least), which is much longer than the actual growth time $\sim l/(u + c) < 0.2$ s. This comparison confirms that diffusive effects can be neglected in the sudden expansion of microscopic bubbles by the frictional soliton.

SI.6. Independence of flow rate saturation on the Reynolds number of the flow

Fig. SI.3 indicates that flow rate saturation (and the bifurcation of the flow from a single-phase low-forcing regime to a two-phase high-forcing regime) is observed at values of the Reynolds number of the flow ($Re \equiv \rho Q/\pi\eta_0 R$) varying by more than two orders of magnitude (including values much lower than one), as the particle volume fraction is varied.

The Reynolds number at saturation $Re_c = \rho Q_c/\pi\eta_0 R$ is actually selected by the shear-thickening onset condition given by Eq. [4] of the main body of the paper, i.e., $Q = Q_c \equiv \pi R^3 \tau_c(\phi)/4\eta_0$. This condition (dashed line in Fig. SI.3) is found to capture the saturated flow rate dependance on both the volume fraction ϕ (main graphics) and the pipe radius R (inset).

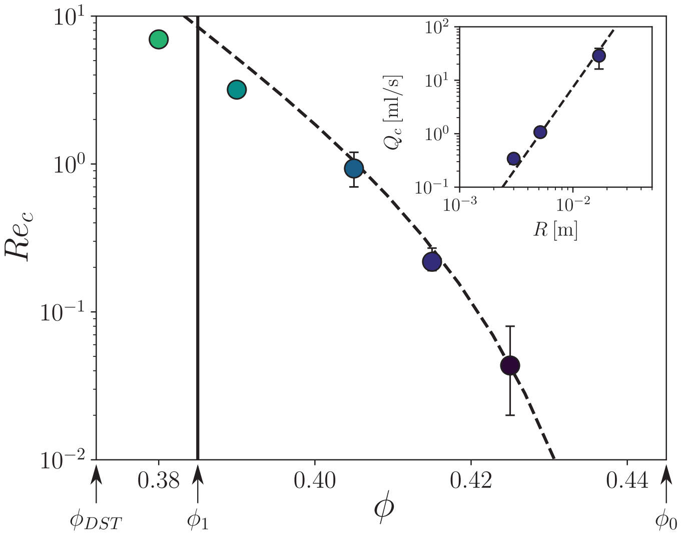

Fig. SI.3. (Main) Reynolds number $Re_c = \rho Q_c/\pi\eta_0 R$ at the onset of flow rate saturation (hence, of the high-forcing regime) vs particle volume fraction ($R = 5.15$ mm, same data as in Fig. 2B of the main body of the paper). (Inset) Saturation flow rate Q_c vs pipe radius ($\phi = 0.415$). The dashed lines are the value expected from Eq. [4] of the main body of the paper, i.e., $Re_c = \rho Q_c/\pi\eta_0 R$, with $Q_c \equiv \pi R^3 \tau_c(\phi)/4\eta_0$.

SI.7. Sampling of the particle volume fraction at the pipe outlet

In order to verify whether the propagation of the frictional soliton is associated, or not, with a significant global redistribution of the particle volume fraction along the pipe, two additional experiments have been conducted, in the low- and high-forcing regimes, respectively, during which a few samples of suspension (~ 10 ml) are collected at the pipe outlet over the drainage duration. The particle volume fraction in each sample is determined by weighing the sample before and after it has been dried, under controlled conditions, in an oven.

Fig. SI.4 presents the evolution of the volume fraction collected at the pipe outlet ϕ_{out} for the low-forcing regime ($\circ$, $\langle \tau_w \rangle / \tau_c(\phi) \approx 0.7$) and the high-forcing regime ($\square$, $\langle \tau_w \rangle / \tau_c(\phi) \approx 2.8$), for the same nominal (prepared) volume fraction of the suspension ($\phi = 0.405$). In both cases the collected volume fraction ϕ_{out} is found to remain undistinguishable (given the experimental accuracy of $\approx \pm 0.5\%$) from the nominal volume fraction ϕ.

This observation indicates that no significant global redistribution of the particle volume fraction along the pipe is associated with the frictional-soliton inception or propagation along the pipe. However, it does not permit to conclude about possibly significant redistribution of the particle volume fraction within the pipe cross-sections.

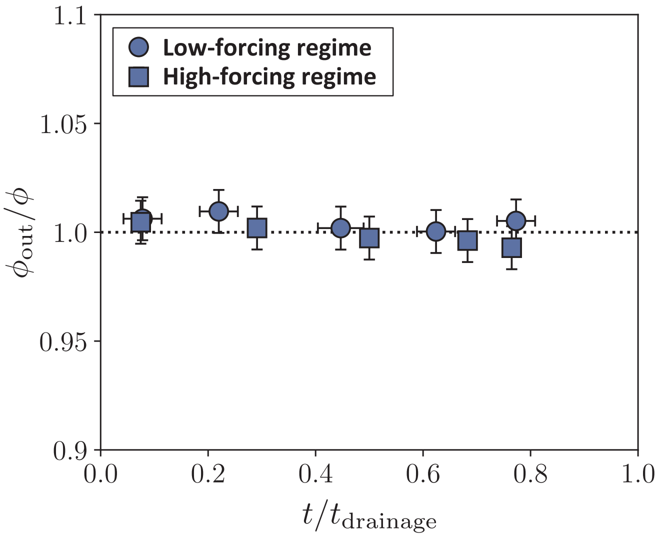

Fig. SI.4. Evolution of the suspension volume fraction ϕ_{out}, as collected at the pipe outlet, vs time. The nominal volume fraction is $\phi = 0.405$. t_{drainage} is the drainage time, at which the suspension free-surface reaches the pipe outlet and about 90% of the total suspension volume has drained. The symbol shape indicates the low-forcing regime ($\circ$, $\langle \tau_w \rangle / \tau_c(\phi) \approx 0.7$) or the high-forcing regime ($\square$, $\langle \tau_w \rangle / \tau_c(\phi) \approx 2.8$). The pipe radius is $R = 5.15$ mm.

SI.8. Main characteristics of the effective rheology and of the frictional soliton for the different shear-thickening suspensions

Table SI.1 lists the low stress effective viscosity and the onset stress of discontinuous shear-thickening obtained from the rheological characterization of the cornstarch suspension and of the four other types of shear-thickening suspensions (A-D) presented in Fig. 6 of the main body of the paper. It also reports the measurements for the flow rate at saturation and the main characteristics of the frictional-soliton flow phase, together with the ranges of particle volume fractions and the range of mean wall stress at which they have been obtained.

			Cornstarch	(A) Potato starch	(B) Cassava starch	(C) Polystyrene spheres + cellulose	(D) Calcite + superplasticizer
Particle vol. fraction	ϕ	(−)	0.380-0.425	0.420	0.448	0.595	0.534
Rheological param.	η_0	(Pa.s)	0.07-0.7	0.20±0.01	0.13±0.01	0.45±0.03	0.3±0.2
	τ_c	(Pa)	2.6-7.6	2.1±0.1	2.8±0.2	23.9±2	0.8±0.3
Mean wall stress	$\langle \tau_w \rangle / \tau_c$	(−)	1.0-31.5	1.3-7.3	2.7-10.9	3.2-5.8	52±31
Saturation flow rate	$Q_c / \frac{\pi R^3 \tau_c}{4 \eta_0}$	(−)	0.8±0.3	1.2±0.1	1.0±0.1	2.6±0.7	4.6±2.1
Frictional-soliton characteristics	$l/2R$	(−)	1.5±0.5	0.9±0.5	1.3±0.3	0.9±0.3	1.1±0.1
	c/U	(−)	1.5±1.0	5.6±1.4	2.5±1.4	0.8±0.2	6.2±0.6
	U_w^{FS}/U_w	(−)	8.2±4.6	5.7±0.4	9.4±1.2	2.0±0.2	8.3±1.0

Table SI.1. Values separated by an hyphen (-) stand for a range of variation. Values separated by a ± symbol indicate the average and the standard deviation over all experiments performed in the range of variation.

Reference

1. G Bossis, Y Grasselli, and O Volkova. Capillary flow of a suspension in the presence of discontinuous shear thickening. *Rheol. Acta*, 61:1–12, 2022.
2. R O'Neill, J Royer, and W Poon. Liquid migration in shear thickening suspensions flowing through constrictions. *Phys. Rev. Lett.*, 123:128002, 2019.
3. M Wyart and M Cates. Discontinuous shear thickening without inertia in dense non-Brownian suspensions. *Phys. Rev. Lett.*, 112:098302, 2014.
4. J Goddard. Material instability in complex fluids. *Ann. Rev. Fluid Mech.*, 35:113–33, 2003.
5. T Divoux, A Fardin, S Manneville, and S Lerouge. Shear banding of complex fluids. *Ann. Rev. Fluid Mech.*, 48:81–103, 2016.
6. F Rocha, Y Forterre, B Metzger, and H Lhuissier. Drag of a shear-thickening suspension on a rotating cylinder. *J. Fluid Mech.*, 970:A35–19, 2023.
7. P Epstein and M Plesset. On the stability of gas bubbles in liquid-gas solutions. *J. Chem. Phys.*, 18:1505–09, 1950.
